# M1 muscarinic receptor is a key target of neuroprotection, neuroregeneration and memory recovery by i-Extract from *Withania somnifera*

**DOI:** 10.1038/s41598-019-48238-6

**Published:** 2019-09-30

**Authors:** Arpita Konar, Richa Gupta, Rajendra K. Shukla, Bryan Maloney, Vinay K. Khanna, Renu Wadhwa, Debomoy K. Lahiri, Mahendra K. Thakur

**Affiliations:** 1 Biochemistry and Molecular Biology Laboratory, Brain Research Centre, Department of Zoology, Banaras Hindu University, Varanasi, 221005 India; 2 CSIR-Institute of Genomics & Integrative Biology, New Delhi, 110025 India; 3 Developmental Toxicology Laboratory, Systems Toxicology and Health Risk Assessment Group, CSIR-Indian Institute of Toxicology Research, Lucknow, 226001 Uttar Pradesh India; 4 Departments of Psychiatry, Stark Neuroscience Research Institute, Indiana University School of Medicine, 320 West 15th Street, Indianapolis, IN-46202 USA; 5 Departments of Medical and Molecular Genetics, Indiana Alzheimer Disesae Center, Indiana University School of Medicine, Indianapolis, IN-46202 USA; 6 DBT-AIST International Laboratory for Advanced Biomedicine (DAILAB), Biomedical Research Institute, National Institute of Advanced Industrial Science & Technology (AIST), Central 4, 1-1-1 Higashi, Tsukuba, Ibaraki 305 8562 Japan; 7 Department of Biochemistry, Autonomous State Medical College, Bahraich, Utter Pradesh 271801 India; 8 Devision of ECD, Indian Council of Medical Research, New Delhi, 110029 India

**Keywords:** Target identification, Alzheimer's disease, Short-term memory, Alzheimer's disease

## Abstract

Memory loss is one of the most tragic symptoms of Alzheimer’s disease. Our laboratory has recently demonstrated that ‘i-Extract’ of Ashwagandha (*Withania somnifera*) restores memory loss in scopolamine (SC)-induced mice. The prime target of i-Extract is obscure. We hypothesize that i-Extract may primarily target muscarinic subtype acetylcholine receptors that regulate memory processes. The present study elucidates key target(s) of i-Extract via cellular, biochemical, and molecular techniques in a relevant amnesia mouse model and primary hippocampal neuronal cultures. Wild type Swiss albino mice were fed i-Extract, and hippocampal cells from naïve mice were treated with i-Extract, followed by muscarinic antagonist (dicyclomine) and agonist (pilocarpine) treatments. We measured dendritic formation and growth by immunocytochemistry, kallikrein 8 (KLK8) mRNA by reverse transcription polymerase chain reaction (RT-PCR), and levels of KLK8 and microtubule-associated protein 2, c isoform (MAP2c) proteins by western blotting. We performed muscarinic receptor radioligand binding. i-Extract stimulated an increase in dendrite growth markers, KLK8 and MAP2. Scopolamine-mediated reduction was significantly reversed by i-Extract in mouse cerebral cortex and hippocampus. Our study identified muscarinic receptor as a key target of i-Extract, providing mechanistic evidence for its clinical application in neurodegenerative cognitive disorders.

## Introduction

Alzheimer’s disease (AD) is the most common form of age-related dementia and is among the most common forms of dementia of any type^1^. AD is primarily distinguished by accumulation of extracellular neuritic plaque that primarily consists of amyloid β peptide (Aβ)^2,3^, by intraneuronal tangles of hyperphosporylated microtubule-associated protein τ (MAPT, or τ)^2,4^, and by synaptic loss^5,6^. All of these processes are interconnected^5,7,8^. The earliest clinical sign that presents is usually loss of cognition and memory^9,10^. Ultimately, before neurodegeneration reaches biologically lethal stages, the person can forget even the closest relatives and be unable to identify them. Emotional costs upon caretakers may be incalculable, as they watch their loved one decline into someone who no longer knows them.

While multiple animal models exist for neuropathology of AD, particularly transgenics that contain one or more autosomal dominant AD mutants, or animals with humanized genes, artifacts of transgene expression may reduce their applicability to reaching conclusions about human diseases^11,12^. Therefore, mechanistic models still have a place in advancing AD research. One such model is to induce amnesia in test animals via scopolamine^13,14^. We have previously worked with this model and find it useful, particularly for mechanistically testing effects of substances with reputed neuroprotective (so-called ‘nootropic’) properties^15,16^.

Impairment in dendrite growth and arborization are cardinal features of memory loss in neurodegenerative diseases^6,17,18^. Associated molecular markers for dendrite growth and arborization include kallikrein 8 (KLK8) and microtubule associated protein2, c isoform (MAP2c), which are pivotal for these processes^19,20^. The MAP2 protein exists in four major isoforms, specifically MAP2a, MAP2b, MAP2c and MAP2d. MAP2a and MAP2b are higher molecular weight isoforms and are the predominant isoforms in the adult CNS. MAP2c and MAP2d, are low molecular weight isoforms. MAP2d contains four repeats in the C-terminus for binding to microtubules whereas MAP2c has three. MAP2c is found in growing dendrites and, in certain populations of neurons, in growing axons. It also is expressed in neurons that have the capacity to regenerate in the adult CNS^21^. MAP2c activity is implicated in AD-associated tauopathy^22^. MAP2c can prevent fibril formation of MAPT^23^. While both MAPT and MAP2c aggregate, their aggregate forms are distinct from each other^24^.

The apolipoprotein E (APOE) ε4 phenotype is associated with increased risk for AD^25–29^. Interestingly, the ε4 phenotype does not bind MAP2c, while the ε3 phenotype, which is not associated with greater AD risk, does^30^. While the dentate gyrus of AD brains produces new neuronal cells, they fail to mature, and this failure is tied to great increase in the MAP2a and MAP2b isoforms of MAP2^31^. In addition, KLK8 deficiency completely impaired the early phase of long-term potentiation^32^.

Multiple modern medications have ‘herbal’ origins, including but not limited to aspirin, digitalin, the biguanide class of antidiabetic drugs, and oncology drugs such as paclitaxel (Taxol). In recent years, herbal remedies from non-’Western’ traditional medicine have gained notice as viable sources for efficacious and safe drugs^33,34^. Among these substances, *ashwagandha* (*Withania somnifera*), aka Indian ginseng, poison gooseberry, or winter cherry, has received recognition as potentially possessing nootropic, neuroprotective, and neuroregenerative activity^15,35–38^.

Inadequate information about mechanisms of action is a stumbling block for regulatory approval of application of many ‘herbal’ substances, including i-Extract, in clinical settings. Therefore, our laboratory previously examined novel neuroprotection and amelioration of memory deficits by *ashwagandha* leaf extract (i-Extract) in scopolamine hydrobromide challenged mice^16^. i-Extract also enhanced expression of neural plasticity genes associated with memory processes^16,39^. However, these studies did not sufficiently explore mechanisms of i-Extract recovery or protection of pathways inactivated by scopolamine or if the mechanism was independent. Therefore, our current goal is to elucidate specific targets of i-Extract that induce both changes in gene expression and in cellular biology.

Scopolamine is a muscarinic acetylcholine receptor (mAChR) antagonist^14^ and has its greatest binding affinity for muscarinic receptors, which are also crucial for plasticity and memory^40,41^. We hypothesized that i-Extract might target the same receptors to exert neuroprotective and neurotrophic activity. Herein, we treated mouse primary neurons and mice with i-extract after challenge with scopolamine. We also used the high-affinity M1 muscarinic receptor antagonist dicyclomine^42,43^ and the muscarinic agonist pilocarpine^44,45^ to investigate neuroprotective and neurotrophic potentials of i-Extract. We measured dendritic formation and growth in primary neuronal cell culture and mouse cerebral cortex and hippocampus. We found that i-Extract treatment reversed adverse effects of scopolamine, and this reversal was mediated through the muscarinic receptor pathway. Dendrite data was echoed by parallel changes in levels of KLK8 and MAP2c. We finally substantiated our findings by muscarinic receptor binding assay, wherein binding of radioligand ^3^H-quinuclidinyl benzilate (^3^H-QNB)^46,47^ was determined in cortical and hippocampal membrane lysates of i-Extract treated mice.

We had previously determined that the active component of i-Extract responsible for anti-scopolamine activity was withanone^16^. Withanone is a steroidal phytocompound that can be extracted from both roots and leaves of *W*. *somnifera*^16,48–51^. A co-occurring withanolide, withaferin, is under investigation for treatment of cancer^52^. Withanone reduced nonspecific cytotoxicity of withaferin without reducing apparent anti-metastatic activity of withaferin^53^. On its own, withanone protected against NMDA-induced cytotoxicity in neuronal cell cultures^54^, suppressed proliferation of activated microglia^48^, decelerated human fibroblast senescence^49^, and improved cognitive function in Wistar rats along with reduction of Aβ^50^. It also recovered scopolamine-induced reduction of activity-regulated cytoskeletal-associated protein (ARC) in mouse hippocampus and associated memory functions^16^. We chose to use i-Extract rather than purified withanone because whole extracts of *W*. *somnifera* are well-tolerated and the field has established activity for the extracts^16,39,49,52,53^. Possible synergistic or antagonistic effects may exist between withanone and withaferin in the context of KLK8 activity, and these will be tested in future experiments.

Our results suggest that the activity of i-Extract in reversing damage of scopolamine challenge operates on the muscarinic acetylcholine receptor pathway, and that this activity is tied to fundamental neuronal cell growth as mediated by MAP2c and KLK8. *Ashwagandha*, as i-Extract, may offer a means to ‘jump start’ cell plasticity in disorders that disrupt memory, such as AD. *Ashwagandha* is not alone in having neurological potential. We have previously noted neurotrophic and neuroprotective properties for the curcuminoids^55,56^ and for aged garlic extract^57^.

## Results

### i-Extract showed remarkable dendrite growth potential in scopolamine-challenged neurons

**Figure 1:**
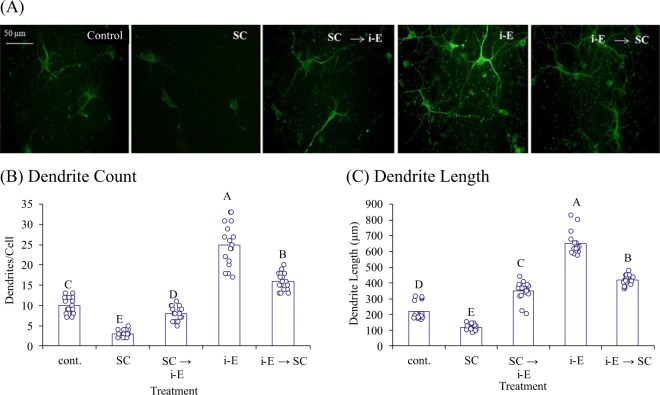
i-Extract shows neuroregeneration and neuritogenic potential. (**A**) Immunocytochemical MAP2 staining of primary hippocampal neurons showing reduced dendrite growth upon treatment with scopolamine (SC) vs. saline control. Notably, i-Extract (i-E) attenuates SC mediated loss in pre- and post treatment conditions. i-Extract also enhances dendrite growth and branching as compared to control. Scale bar represents 50 μm. Charts represent (**B**) average number of dendrites or (**C**) dendrite length in each treatment group. Letters represent pairwise differences (p ≤ 0.05) all vs. all (FDR). Samples sharing a letter do not significantly differ.

**Figure 2:**
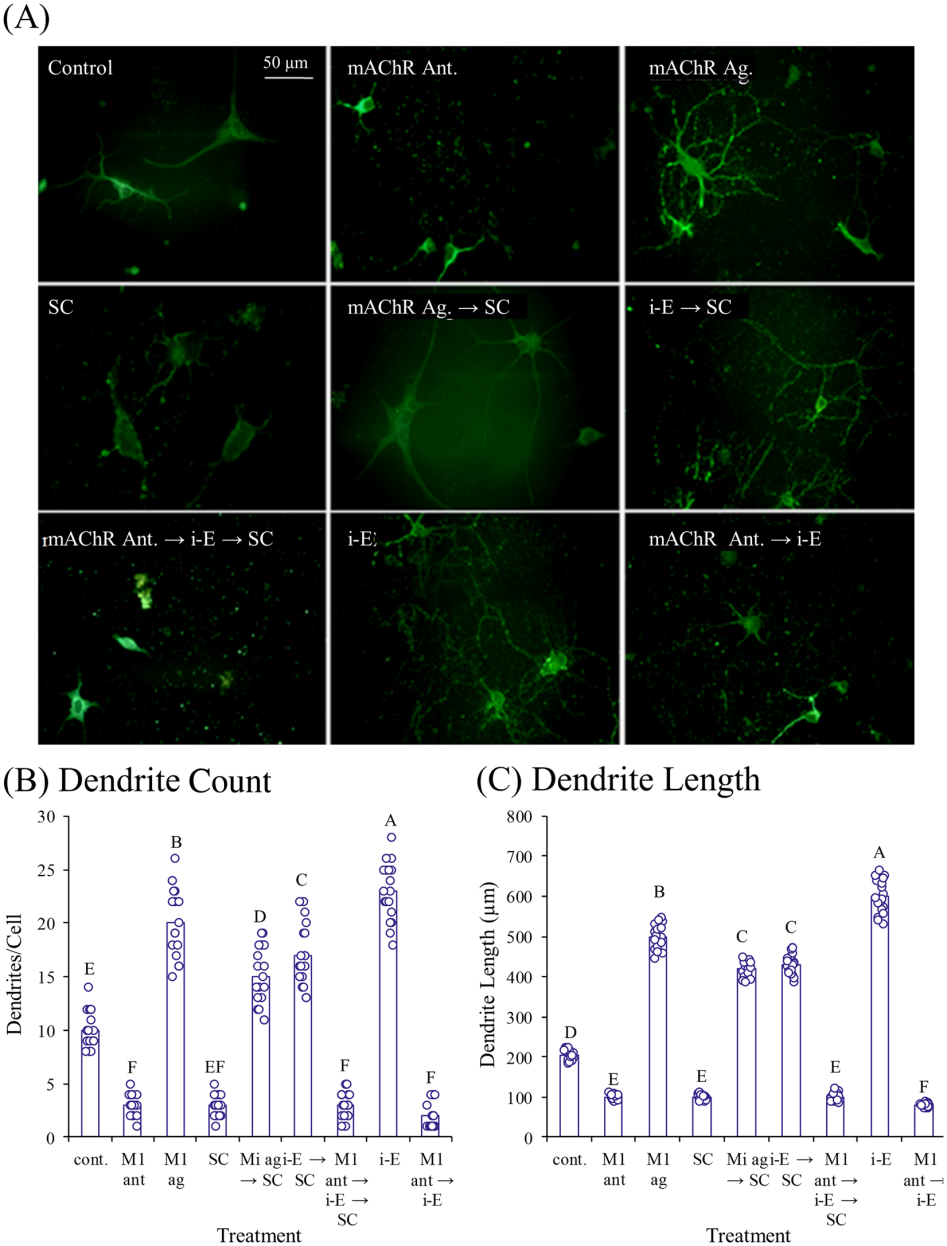
MAP2 staining shows dendrite growth enhancement via i-Extract (i-E). (**A**) Treated neuron photomicrographs with immunocytochemical MAP2 staining. Enhancement of (**B**) number of dendrites of (**C**) dendrite length via i-E or mAChR ag are not only eliminated in kallikrein 8 (KLK8) siRNA silenced hippocampal neurons but are lower than controls. Levels are presented relative to saline controls. Letters indicate statistical groups by FDR (p ≤ 0.05).

We analyzed the effect of i-Extract on scopolamine-damaged neurons in both pre- and post- treatment regimes. As anticipated, scopolamine treatment caused drastic loss in average dendrite number (−70%) and length (−48%). Pretreatment with i-Extract markedly attenuated scopolamine-induced reduction, and post-treatment regenerated the lost dendrites. i-Extract alone exhibited neuritogenic potential by increasing dendrites +150% and dendrite length + 195% compared to control (Fig. 1). We determined molecular correlates of dendritogenesis by i-Extract. *In-vitro* gene silencing confirmed that KLK8 and MAP2 proteins are involved in neuritogenic property of i-Extract. i-Extract did not induce length and arborization of MAP2 positive dendrites upon KLK8 knockdown (Fig. 2).

### Muscarinic receptor modulators altered i-Extract induced dendrite growth and expression of molecular markers

**Figure 3:**
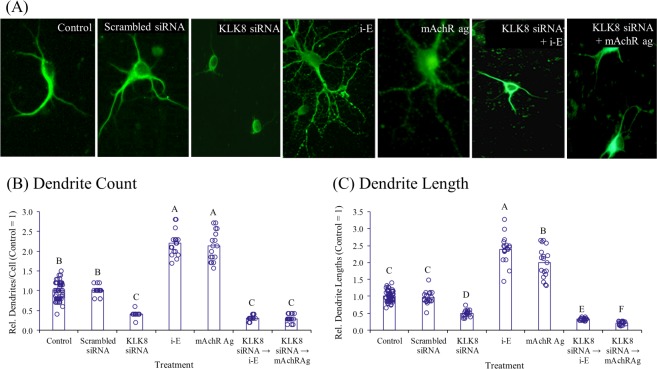
MAP2 stained primary hippocampal neurons show effects of KLK8 knockdown for muscarinic agonist (mAChR-Ag) treatment. (**A**) Knockdown of KLK8 by siRNA drastically reduced dendrite length and arborization and inhibited dendritogenic potential of i-E mAChR-Ag replicated effects of i-E in dendrogenesis and dendrite outgrowth. Column charts represent (**B**) average number of dendrites or (**C**) dendrite length in each treatment group. Letters indicate statistical groups by FDR (p ≤ 0.05). Treatments sharing letter (within brain region) do not significantly differ.

We analyzed effects of the muscarinic antagonist dicyclomine and agonist pilocarpine singly and in combination with scopolamine and i-Extract. Muscarinic blockade by dicyclomine caused loss of dendrites, comparable to damage by scopolamine. Pre-treatment with dicyclomine also profoundly inhibited dendritogenesis by i-Extract. On the contrary, muscarinic agonism by pilocarpine augmented dendrite growth and inhibited damage by scopolamine. Interestingly, muscarinic activation-mediated changes in dendrite morphology was almost completely abolished in KLK8 compromised hippocampal neurons (Figs 2–3)

### i-Extract attenuated scopolamine inhibition of binding of muscarinic radioligand

A significant decrease in the binding of ^3^H-QNB was observed both in cerebrocortical (55%) and hippocampal (41%) membranes of scopolamine-exposed mice compared to controls (Table 1). Alteration of binding was due to a significant decrease in affinity, as reflected by higher K_d_, with no significant change in binding sites (B_max_) in either brain region as revealed by Scatchard analysis (Table 2). Interestingly, administration of i-Extract attenuated scopolamine-induced decrease in the binding of muscarinic receptors both in the cerebral cortex (88%) and hippocampus (34%) (Table 1). No significant effect on binding of cholinergic–muscarinic receptors was observed in either of the brain regions of mice exposed to i-Extract alone as compared to control group.

**Table 1:** Effect on ^3^H-QNB binding to hippocampal and cerebrocortical membranes of mice following exposure to scopolamine, i-Extract or their co-exposure.

Brain Region	Control	n	Scopolamine^a^ (% decrease)^a^	n	i-Extract	n	Scopolamine + i-Extract^b^ (% attenuation)	n
Cerebral cortex	557 ± 45	5	250 ± 41^*^ (55%)	5	506 ± 37	4	470 ± 35* (88%)	4
Hippocampus	711 ± 40	4	419 ± 62^*^ (41%)	4	662 ± 35	5	564 ± 29* (34%)	4

^a^ compared to control group.

^b^ compared to scopolamine exposed group.

*p ≤ 0.05.

**Table 2:** Scatchard analysis of ^3^H-QNB in cerebrocortical and hippocampal membranes of mice following scopolamine and/or i-Extract.

	Cerebral Cortex				Hippocampus			
	Control	Scopolamine^a^	i-Extract	Scopolamine + i-Extract^b^	Control	Scopolamine^a^	i-Extract	Scopolamine + i-Extract^b^
*K*_*d*_	0.57 ± 0.07	^†^1.19 ± 0.12	0.68 ± 0.06	^†^0.73 ± 0.08	0.67 ± 0.05	^†^1.31 ± 0.11	0.74 ± 0.08	^†^0.86 ± 0.05
*B*_*max*_	943 ± 58	861 ± 66	901 ± 86	991 ± 81	1133 ± 121	1048 ± 84	1171 ± 98	1110 ± 72

^a^K_d_ is compared to control group.

^b^K_d_ is compared to scopolamine exposed group.

^†^p ≤ 0.01.

### i-Extract upregulated important protein markers, KLK8 and MAP2 in scopolamine treated mice

**Figure 4:**
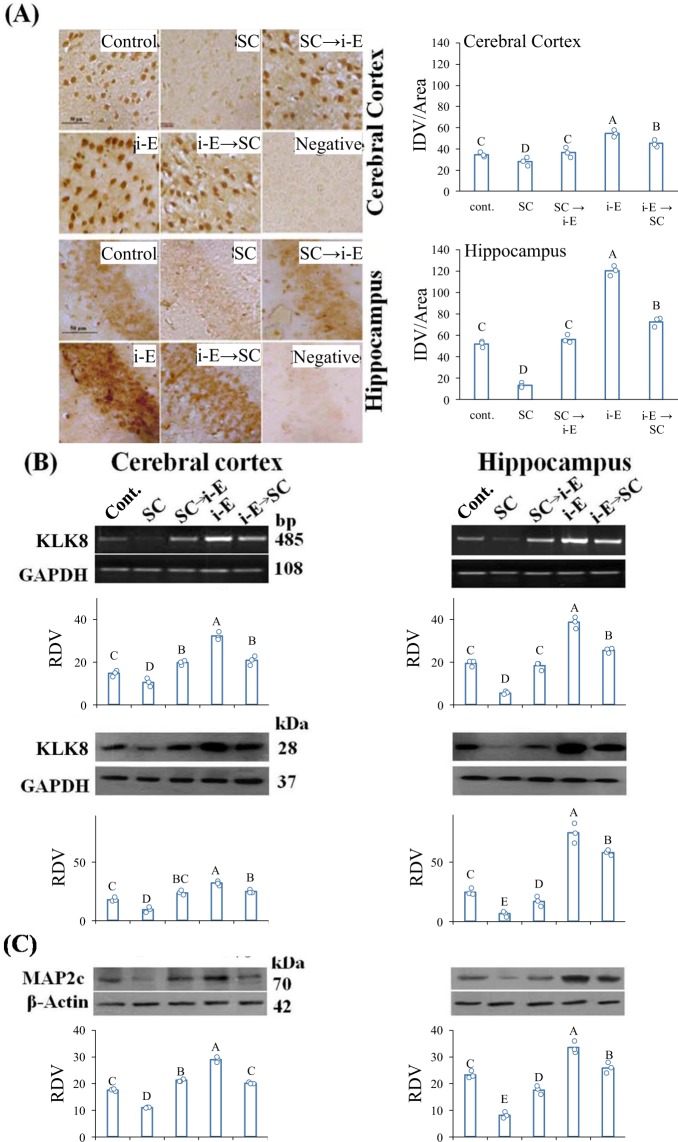
Cortical and hippocampal levels of dendrite growth markers, KLK8 and MAP2 are reduced in scopolamine treated mice but drastically upregulated by i-E. (**A**) *In situ* hybridization analysis of KLK8 mRNA in cerebral cortex and hippocampus. Photomicrographs are captured at 400x magnification and scale bar represents 50μm. Column chart represents IDV/area of KLK8 expression. Letters indicate statistical groups by FDR (p ≤ 0.05). Treatments sharing letter (within brain region) do not significantly differ. Negative-Unlabeled probe. (**B**) RT-PCR and western blot analyses of KLK8 mRNA and protein respectively and (**C**) MAP2 protein in cerebral cortex and hippocampus. Column chart represents RDV of KLK8 (IDV of KLK8/GAPDH) and MAP2 (IDV of MAP2c/β-Actin) expression.

i-Extract also altered KLK8 and MAP2 levels *in vivo* in cerebral cortex and hippocampus of scopolamine (SC)-treated mice. RNA *in situ* hybridization showed significant reduction of KLK8 mRNA in both cerebral cortex and hippocampus of SC-treated mice. KLK8 depletion was attenuated by i-Extract in both brain regions in pre-treatment and post-treatment regimens. i-Extract per se also markedly elevated endogenous (saline control) KLK8 level (Fig. 4A). Specific percent changes are detailed in Supplementary Table 1. Apparent differences between response in cortex and hippocampus were likewise, significant by two-way ANODE (Supplementary Table 2).

Consistent with the *in situ* data, quantitative reverse transcription polymerase chain reaction (RT-PCR) analysis demonstrated cortical and hippocampal KLK8 mRNA decrease in SC-treated mice. Notably, i-Extract treatment recovered the reduced KLK8 mRNA level in pre- and post-treated groups. Endogenous KLK8 was also up-regulated (2-fold) by i-Extract in both brain regions. KLK8 protein level was reduced in cortex and hippocampus of SC-treated mice. Interestingly, i-Extract increased KLK8 protein level in SC-treated and control mice (Fig. 4B). Specific percent changes are detailed in Supplementary Table 1. The apparently greater extent of response in hippocampus vs. cortex was significant by two-way ANODE (Supplementary Table 2).

MAP2 protein showed similar expression patterns, being reduced in cerebral cortex of the SC-treated mice. i-Extract increased MAP2c level in SC-treated as well as control mice. In hippocampus, the protein level was significantly reduced in SC-treated mice and showed recovery following pre and post-treatment with i-Extract. i-Extract caused increase in hippocampal MAP2c level of control mice (Fig. 4C, Supplementary Table 1). Apparent differences between responses in cortex and hippocampus were significant.

**Figure 5:**
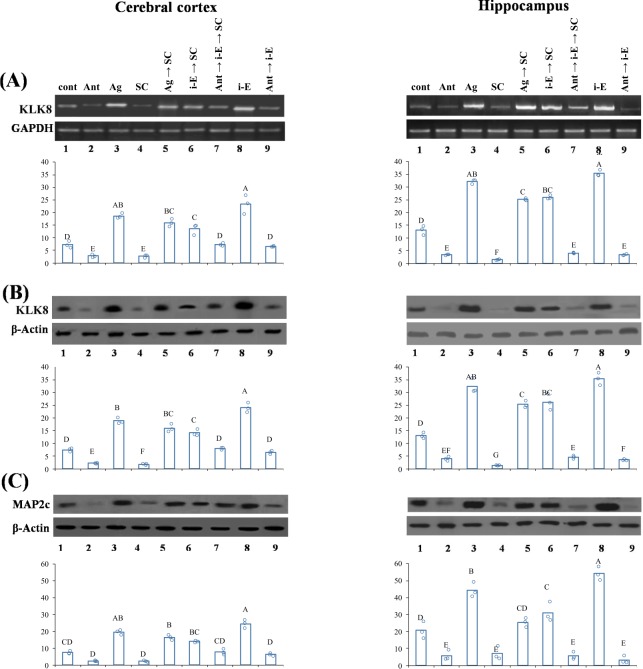
Muscarinic receptors regulate i-Extract mediated increase in KLK8 and MAP2 expression. M1 agonist (mAChR- Ag) inhibited SC mediated decrease and caused up-regulation while antagonist (mAChR- Ant) eliminated i-E mediated increase and caused down-regulation of (**A**) KLK8 mRNA and (**B**) KLK8 protein and (**C**) MAP2c protein in cerebral cortex and hippocampus. Column chart represents RDV (IDV of KLK8/GAPDH; IDV of KLK8/β-actin; IDV of MAP2c/β-Actin). Letters indicate statistical groups by FDR (p ≤ 0.05). Treatments sharing letter (within brain region) do not significantly differ.

We checked expression of KLK8 and MAP2 after M1 antagonist and agonist treatment combined with scopolamine and i-Extract. M1 antagonist decreased (−61%) and agonist increased (+157%) KLK8 mRNA in cerebral cortex. Pre-treatment with M1 agonist completely inhibited scopolamine-induced decrease in KLK8 mRNA and further increased it by 5-fold as compared to scopolamine. M1 antagonist inhibited KLK8 upregulation by i-Extract. In hippocampus, the effects of both antagonist and agonist were more drastic than in cerebral cortex. Complete comparisons of treatment results are presented in Supplementary Table 3 (Fig. 5A).

KLK8 protein was reduced by M1 antagonist and increased by agonist compared to saline in cerebral cortex. KLK8 protein expression was increased by pre-treatment with agonist compared to scopolamine, but reduced by antagonist, compared to i-Extract. In case of hippocampus, KLK8 protein level was significantly reduced by antagonist and enhanced by agonist. Pre-treatment with agonist increased KLK8 protein expression compared to scopolamine. Treatment with antagonist prior to i-Extract reduced expression of KLK8 protein (Fig. 5B, Supplementary Table 3).

**Figure 6:**
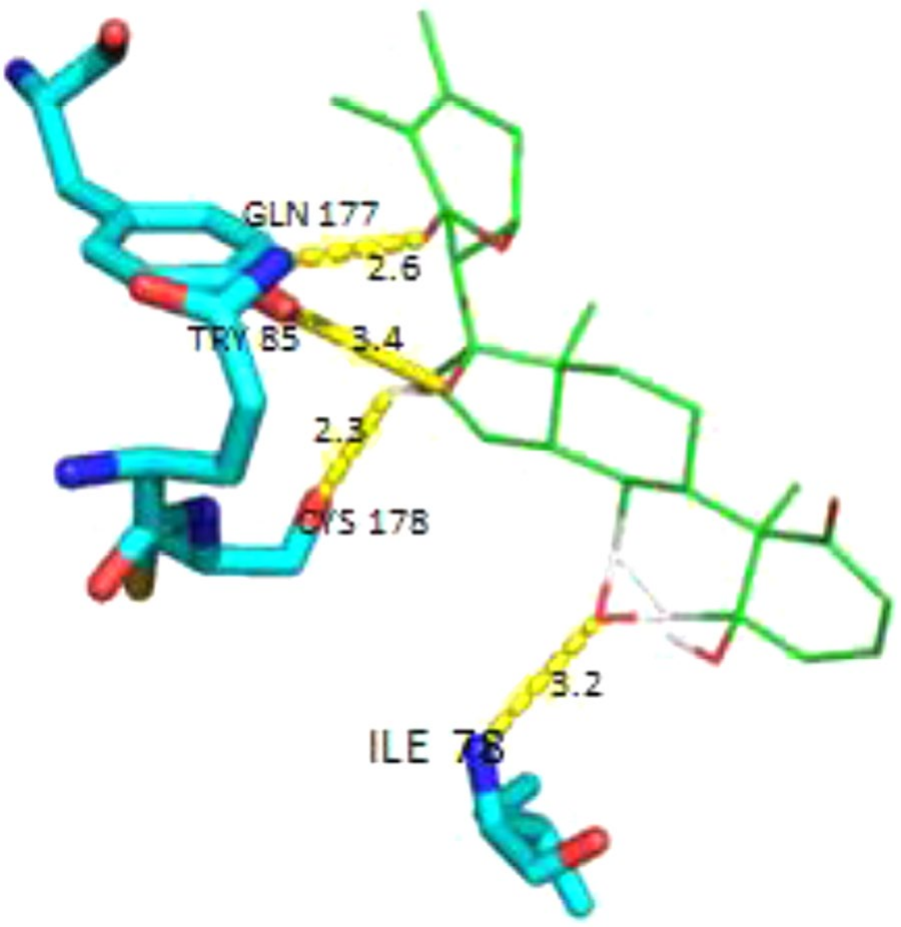
Docking analysis of withanone to M1 receptor molecule. Docking analysis was done with the AutoDock Tools (http://autodock.scripps.edu/). The structure description for withanone (https://pubchem.ncbi.nlm.nih.gov/compound/21679027) was obtained from PubChem and for the M1 receptor (http://www.rcsb.org/structure/5CXV) from the RCSB protein data bank. These were used to perform a docking analysis. Bonds were predicted (yellow lines) between withanone and four amino acids in the M1 receptor protein molecule.

MAP2 was also reduced by M1 antagonist and increased by M1 agonist in cerebral cortex. Both MAP2 decrease by scopolamine and upregulation by i-Extract were inhibited by agonist and antagonist, respectively. In the hippocampus, the effects of both M1 antagonist and agonist were more pronounced than in cerebral cortex. M1 antagonist downregulated MAP2c level, and agonist increased MAP2c level as compared to saline. Moreover, M1 antagonist inhibited the effect of i-Extract, and agonist blocked the effect of scopolamine (Fig. 5C, Supplementary Table 3). Apparent differences of response in hippocampus vs. cortex was significant by two-way ANODE (Supplementary Table 4). Finally, docking analysis of withanone to the M1 receptor amino acid sequence predicted that withanone would bind with high affinity (ΔG = −10.1) to the ILE 78, TRY 85, GLN 177, and CYS 178 of the M1 receptor protein (Fig. 6)

## Discussion

Previous studies demonstrated neuroprotective and neurotrophic potential of *ashwagandha* extracts in diverse cellular and animal models of neurodegeneration and cognitive decline, particularly those for AD^58–63^. Our own earlier reports highlighted the neuroprotective action of a well-characterized extract of *ashwagandha* (i-Extract), including induction of multiple cellular and molecular alterations in a scopolamine model of memory loss^16,39^. We extend our previous work to expose and explain the upstream ‘master switch’ i.e., the prime biological target that drives i-Extract activity in brain. Our work is significant in that it establishes a foundation for formal therapeutic value and clinical application. The present study elucidates key target(s) of i-Extract via cellular, biochemical, and molecular techniques in a relevant animal amnesia model and primary hippocampal cultures. We specifically focused on modulation of neuroarchitecture including rebuilding neuronal networks and neuritogenesis, central processes for brain plasticity and higher order functions of memory and cognition.

As scopolamine is a cholinergic blocker having maximum affinity to muscarinic receptor subtypes, we speculated that i-Extract might operate through the same pathway. Our previous reports showed that pre-treatment of i-Extract was more effective than post-treatment, which strengthened this hypothesis. As we expected, a muscarinic receptor antagonist, dicyclomine, completely abolished the ability of i-Extract to induce neurite growth and expression of marker proteins. On the other hand, the muscarinic agonist pilocarpine simulated nootropic action of i-Extract in recovery of scopolamine insults as well as neurite enhancement. We propose, therefore, that among its potentially many actions, components of i-Extract should be further explored as muscarinic agonists. Of particular note, our cytological and biochemical studies are supplemented with behavioral data; specifically that i-Extract protects mice from the memory-damaging properties of scopolamine, *reverses* the effects of scopolamine, and *improves upon* control animal performance in the Morris Water Maze^39^. Given *ashwagandha*’s long use in a large population, we speculate that it may be a safer, but effective, alternative to currently available medicinal muscarinic agonists.

Cholinergic neurotransmission via muscarinic receptor downstream signaling is particularly important in neuronal excitability, plasticity and cognitive function^64^. Dysfunctional muscarinic signaling contributes to the pathophysiology of memory impairment during aging and neurodegenerative disorders such as AD, but the molecular correlates are not fully understood^65^. Earlier studies reported that cholinergic agonists like carbachol and pilocarpine induced neuronal differentiation and dendritic growth^66^ and mRNA expression of synaptic plasticity genes such as brain derived neurotrophic factor (BDNF), nerve growth factor (NGF)^67^ and ARC^68^ in rat hippocampus. Some recent studies have shown that the cholinergic hypofunction induced by the toxin ^192^IgG-saporin impaired memory acquisition, possibly through hippocampal ARC and BDNF down-regulation via muscarinic receptors^68^. Upon acetylcholine binding, muscarinic receptor binds Gαq-proteins to activate phosphatidylinositol hydrolysis, calcium influx and subsequently cascade of kinases that include protein kinase C (PKC) and mitogen activated protein kinase (MAPK), which phosphorylate transcription factors and induce gene expression^45^. It is noteworthy that diminished phosphorylated-cAMP-responsive element-binding protein (pCREB) activity in the prefrontal cortex is associated with AD^69^, that Aβ42 plays a role in this loss of function, and that resveratrol reverses this deficit^70^. Our muscarinic radioligand binding assay corroborates this hypothesis, as we observed increased binding of ^3^H-QNB to the receptors as compared to scopolamine. However, kinases and transcription factors in i-Extract induced muscarinic signaling need to be investigated.

Muscarinic receptors have distinct subtypes (M1-M5), amongst which, M1 receptor expression is predominant in frontal cortex and hippocampus, and their role in cognition is well documented in animal and human studies^71^. The muscarinic antagonist dicyclomine that we used is M1 selective, although the agonist pilocarpine has affinity to other receptors as well. Therefore, it is likely that i-Extract has preference for the M1 subtype, and our observations support this conclusion. Our earlier reports agree with the present work: Effect of i-Extract is more pronounced in hippocampus than in cerebral cortex. M1 receptor knockout mice are deficient in hippocampal dependent memory processes^72^, and the M1 antagonist dicyclomine impaired hippocampal integrity, but the hippocampus independent functions were unaffected^73^. M1-regulated kinases, particularly PKC, are more abundant in hippocampus than in cerebral cortex^74,75^. Therefore, we conclude that i-Extract induction of M1 signaling would be stronger in hippocampus. An M1 specific ligand binding assay would confirm this.

**Figure 7:**
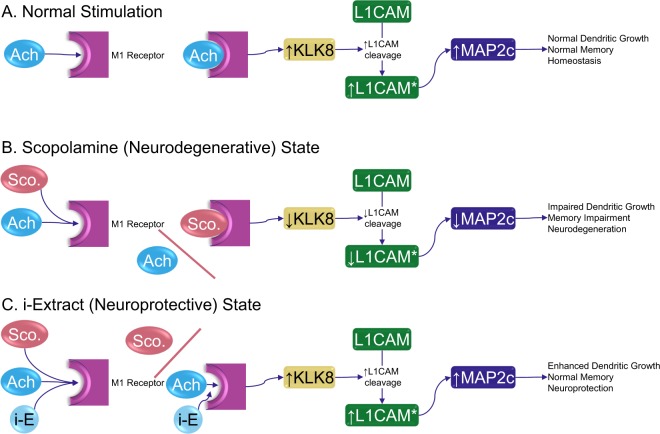
Proposed pathway for muscarinic targeting by i-Extract and promotion of dendrite growth. (**A**) Normal acetylcholine stimulation of dendritic outgrowth and memory. Acetylcholine (Ach) binds to M1 receptors in a stimulatory fashion. Downstream signaling upregulates KLK8 production. KLK8 cleaves L1CAM. Mature L1CAM drives preferential expression of MAP2c, which stimulates dendritic growth and normal memory. (**B**) Scopolamine disruption. Scopolamine inhibition of M1 receptor signaling disrupts dendritic health and memory through inhibition of KLK8 production. (**C**) i-Extract neuroprotection. i-Extract can exclude scopolamine, but beyond this, it stimulates activity of the M1 receptor in some fashion, either through acting agonistically on Ach binding or a more direct mechanism of i-E components. This stimulation then associates with increased expression of synaptic proteins like KLK8. KLK8 cleaves L1CAM, which enhances specific production of MAP2c, thus enhancing dendrite growth.

The M1 receptor turns out to be a key biological target of i-Extract, according to a model that uses both scopolamine and an M1-specific antagonist. Our work, herein, demonstrates that i-Extract stimulates muscarinic receptors, reverses scopolamine blockade of these receptors and that of specific antagonists such as dicyclomine. It further protects against scopolamine. Administration of i-Extract further increases expression of neurite growth proteins such as KLK8 and MAP2c, which then could facilitate healthy dendrite morphology. We propose a simple, explicit model based on KLK8 activity (Fig. 7). Under normal conditions, acetylcholine binds M1 receptors, stimulating KLK8 production, which cleaves L1 cell adhesion molecule (L1CAM). Activated L1CAM stimulates MAP2c, which leads to normal dendritic growth and memory (Fig. 7B). Under neurodegenerative conditions (such as scopolamine inhibition at the M1 receptor), KLK8 levels are not stimulated. This leads to reduced cleavage of L1CAM to its active state. L1CAM levels are correlated to production of MAP2c^20,76^, specifically over other MAP2 moieties. Insufficient MAP2c leads to impaired dendritic growth, memory impairment, and neurodegeneration (Fig. 7B). However, in the presence of i-Extract, KLK8 levels are enhanced, both in comparison to scopolamine reduction *and* compared to unstimulated cultures and tissues, resulting in greater cleavage of L1CAM, producing more MAP2c, and ending in enhanced/restored dendritic growth and neuroprotection (Fig. 7C). It is noteworthy that we showed more than mere reversal *and blockade* of scopolamine effects. Instead, stimulation by i-Extract brought about activity that *exceeded* control samples, indicating that i-Extract is both neuroprotective *and* neurotophic.

We showed herein that i-Extract could regenerate scopolamine damaged neurites and enhance existing neuronal networks by upregulating the neurite growth markers KLK8 and MAP2. We also recently demonstrated that KLK8 inhibition alters processing of L1CAM and induces deficiency in dendrite arborization of mouse primary brain cell cultures^77^. This appears to be in stark contradiction to reports of greater than 11-fold elevation of KLK8 mRNA in AD patients^19^. KLK8 was elevated in brains of female transgenic (CRND8) mice, vs. males, although both males and females develop AD-like symptoms and in brains of both AD and non-AD women^78^. Finally, inhibiting KLK8 in mouse models improved both pathology and cognitive function in transgenic mice^79^. Interestingly enough, two substrates of KLK8 (ephrin receptor B2 and FKBP prolyl isomerase 5) were significantly elevated in early AD stages in the same study, although their levels diminished as KLK8’s increased. Oddly enough, KLK levels were elevated in the transgenic mice but *diminished* as the mice progressed through the transgenic AD-like pathology. Thus, while KLK8 blockade may ameliorate symptoms in a mouse model, that model did not have a KLK8 expression profile typical of human AD progression. We cannot consign KLK8 to the “bad molecule bin”. For example, KLK8 deficiency impairs gamma oscillations in the hippocampus, which impairs memory^80,81^. We propose that KLK8 may need to exist in a “Goldilocks zone”, between deficiency and excess, and that some amnestic conditions are conditions of deficiency. It is not excessively novel to propose that specific molecules may have effects of both deficiency and excess, such has been proposed for fatty acid metabolism^82^ and oncology treatments^83^.

In short, more explicit study of the *staging* of KLK8 levels vs. AD needs to be investigated. Under the current paradigm, even “early” AD is a “late” stage of a long-prodromal condition. Elevation of KLK8 in the AD brain may reflect an attempt to repair AD-related damage already done. Unfortunately, proxy tissues for brain are not convenient. Exploring CSF KLK8 levels in middle-aged and aging subjects who are cognitively normal, suffering from subjective memory complaint, from MCI, and from AD (early and late-onset type) may help elucidate KLK8’s broader role, much as CSF studies have been valuable in understanding ratios of phosophorylated τ to total τ, and Aβ42/Aβ40 ratios in AD. To do so would also require measuring levels of KLK8 substrates and processing enzymes, to provide further context.

Most on-the-market treatment approaches for AD attempt to reduce deficiencies in cholinergic transmission via cholinesterase inhibitors. The M1 muscarinic receptor is also under investigation as an AD drug target^84^. In this regard, our work provides a mechanistic insight towards therapeutic application of M1 stimulants, such as i-Extract, in neurodegeneration and other cognitive disorders, though detailed pathway analysis is still necessary.

In a broader context, memory loss is one of the most tragic symptoms of AD. A person essentially disappears slowly before their loved ones. Treatments of causation are, certainly, to be sought after, but the field has been moving to a consensus that any cause-based treatments for AD would have to be administered at earlier and earlier points^2,85–87^. Eventually, a logical progression might indicate that ‘at risk’ individuals, with no actual symptoms, could be shackled to potentially expensive pharmaceuticals, with unknown long-term side effects. Medicine would have regressed to a state of ‘ritual administration’ of treatments, never daring to take someone off, due to the fear that the person *might* develop a disease such as AD at a later stage in life.

Some preventative suggestions do not go to such excess, particularly those that emphasize improvements in diet^88–91^ or exercise^92–95^. Others have investigated potential benefits of dietary supplementation^96–99^. Use of traditional ‘medicinal’ herbs such as *ashwagandha* would fall into the last category. However, all of these methods require people who might or might not see themselves as at risk for dementia, several decades down the road, to exercise restraint and foresight that are praiseworthy but are also, unfortunately, all too rare. It may be practical to combine palliative and supportive medication with treatment of cause. That is, one would see new-generation drugs, such as inhibitors of the enzymes that produce Aβ peptide from its precursor, not as *replacements for* symptomatic relief, but as *partners to* symptomatic relief. That is, supportive care would prevent further deterioration of an AD patient, while the enzymatic inhibitors would facilitate the slower process of ‘cleanup’ of unwanted aggregates and subsequent neuroregeneration and rearboraization (perhaps also pharmaceutically assisted). If these supportive substances were not merely neuroprotective but also neurotrophic, the combination would have a ‘best chances’ outcome of not only permanently halting disease but potentially reversing it. Our studies suggest that *ashwagandha* may offer such a unique possibility.

## Methods

### Animals

Male Swiss albino strain mice (8 ± 1 weeks) from an inbred colony were used for the study. Females were excluded to prevent estrous cycle effects, which would have required synchronization. Animal handling and experiments were conducted in accordance with the guidelines of the Institutional Animal Ethical Committee and the Central Animal Ethical Committee, Banaras Hindu University (BHU), Varanasi, India. All experimental protocols involving Swiss albino strain mice were approved by the institutional animal ethical committee of faculty of science of BHU, Varanasi.

### Primary culture of mouse hippocampal neurons

Hippocampal neurons were prepared from 0-day old neonatal mice. Briefly, pups were decapitated; hippocampi dissected out, minced, trypsin digested (0.25% trypsin, Invitrogen), and single cell suspension prepared by vigorous trituration. The pellet was resuspended in complete neurobasal medium containing 2% B27 supplement and 2 mM GlutaMax (Invitrogen). Cells were seeded at a density of 2.5 × 10^5^ cells/ml of complete medium in poly-l lysine coated culture plates and kept at 37 °C and 5% CO_2_ in a humidified CO_2_ incubator.

### KLK8 knockdown

Primary hippocampal neurons were transfected with KLK8 specific siRNA (Ambionsi1138455; 50 nM) using Lipofectamine® RNAiMAX transfection reagent according to the manufacturer’s protocol. After 48 h of transfection, cells were harvested for western blotting and immunocytochemistry experiments.

### Drugs and treatments

Desiccated alcoholic extract of *ashwagandha* (*Withania somnifera*) leaves (i-Extract^16^) was dissolved in 0.5% dimethyl sulfoxide (DMSO) (vehicle) and orally administered at 200 mg/kg BW to mice. All other treatments were administered by i.p. injection. However, all mice received the same overall handling. Animals not administered i-Extract were given 0.5% DMSO orally by gavage, and animals not administered injection drugs were nevertheless injected with the same volume of 0.9% saline. *In-vivo-* Scopolamine hydrobromide (Sigma-Aldrich, USA) dissolved in 0.9% saline (vehicle) was administered to mice (3 mg/kg BW) and equal volume of saline to control animals. The M1 receptor antagonist dicyclomine hydrochloride (10 mg/kg BW) and agonist pilocarpine hydrochloride (50 mg/kg BW) were dissolved in 0.9% saline and injected alone or 2 h prior to i-Extract and scopolamine, respectively. Drugs were administered for 7 days, mice were sacrificed and brain regions (cerebral cortex and hippocampus) were dissected out for the cellular, molecular and biochemical assays. Animals dosed with both i-Extract and scopolamine were dosed with a one-hour interval between different drugs. Specific dose regiments are summarized in Supplementary tables 5 and 6. For all experiments, the brain regions of three animals were pooled to produce three pools, due to hippocampus size.

### Tissue cultures

Respective tissue-derived cell cultures (60–70% confluent) grown for 5 days were treated with respective drugs. Scopolamine (3 mM) and i-Extract (1 µg/ml) were added to the cells for 3 h and 24 h, respectively, and then culture medium was replaced with the fresh medium. Dicyclomine (3 mM) and pilocarpine (1 mM) were added to the cells for 2 h. For KLK8 knockdown experiments, 3 days grown cells were transfected with KLK8 siRNA and post 48 h treated with i-Extract for another 24 h or pilocarpine for 2 h.

### RT-PCR

RNA isolated from cerebral cortex and hippocampus of different experimental groups was first reverse transcribed into cDNA using reverse transcriptase. The cDNA was used as a template for subsequent semi-quantitative PCR amplification using specific primers for KLK8^20^ and GAPDH.

### Western blotting

Cerebral cortical and hippocampal lysates (40 μg) were used for western blotting using conventional methods. The primary antibodies (anti- KLK8 M-51 sc-29234 goat polyclonal antibody, Santa Cruz biotechnology; anti-MAP2 M9942 mouse monoclonal antibody, Sigma; anti-GAPDH, sc-137179, mouse monoclonal antibody Santa Cruz biotechnology; β-actin A3854 mouse monoclonal antibody, Sigma) and secondary antibodies were used at adequate dilutions (goat-anti-rabbit IgG HRP, 1:2000 for KLK8 and goat anti mouse 1:3000 for MAP2 and goat-anti-mouse IgG HRP, 1:2000 for GAPDH). While our MAP2 antibodies detect all major isoforms of the protein, we distinguished the MAP2c band by molecular weight (70 kDa for MAP2c vs. 280 kDa for MAP2a and MAP2b).

### Immunocytochemical analysis of dendrite growth

Cells grown on glass coverslips were washed with 1x phosphate buffered saline (PBS); and fixed with pre-chilled methanol: acetone (1:1 v/v) for 5–10 min at room temperature. Fixed cells were permeabilized with 0.32% Triton X-100 in PBS for 15 min, and blocked with 5% goat serum in 1xPBS for 1 h. Cells were incubated with mouse monoclonal anti-MAP2 antibody (Sigma) at 4 °C for 24 h, washed thrice with 0.1% Triton X-100 in 1xPBS (PBST) for 5 min each and incubated with fluorescein isothiocyanate (FITC) conjugated goat anti mouse secondary antibody (Sigma). After three washings with PBST for 5 min each, coverslips were mounted on glass slides using 4′,6-diamidino-2-phenylindole (DAPI) mounting medium. Cells were visualized by Leica inverted fluorescence microscope (Leica DFC 450C).

### Muscarinic receptor radioligand binding assay

Muscarinic receptor radioligand binding assay was carried out in mouse cerebral cortex and hippocampus. Crude synaptic membrane fraction was prepared by homogenizing the tissues in Tris–HCl buffer (5 mM, pH 7.4) and centrifuged (40,000 × g) for 15 min at 4 °C. The pellet was suspended in homogenization buffer (5 mM Tris–HCl, pH 7.4) and again centrifuged (40,000 × g) for 15 min at 4 °C. The pellet obtained was finally suspended in Tris–HCl buffer (40 mM, pH 7.4) and stored at −20 °C. For the binding assay, membrane protein fractions were incubated with ^3^H-quinuclidinyl benzilate (^3^H-QNB, 1 × 10^−9^ M), for 15 min at 37 °C. A set of tubes containing atropine sulfate (1 × 10^−6^ M), a competitor, was also run simultaneously to assess nonspecific binding. Soon after incubation, the contents of the binding tubes were rapidly filtered on glass fiber discs (25-mm diameter, 1.0-μm pore size; Whatman GF/B). The filter discs were washed twice rapidly with cold Tris–HCl buffer (40 mM) to remove unbound radioligand. They were then dried and counted in 5 ml of scintillation mixture (PPO, POPOP, naphthalene, toluene and methanol) with a β-scintillation counter at an efficiency of 30–40% for ^3^H. Specific binding was calculated by subtracting the nonspecific binding (in the presence of atropine sulfate) from the total binding and expressed as picomoles of ligand bound per gram protein. Scatchard analysis was carried out using different concentrations of ^3^H-QNB to determine whether change in the binding is due to alteration in the affinity (K_d_) or number of receptor binding sites (B_max_).

### Data capture and analysis

For *in vivo* studies, each experiment was repeated three times (n = 9 mice/group) and tissue samples pooled in three-mouse groups before assays. For *in vitro* studies, treatments were performed in three independent culture dishes, and the experiment was repeated three times. To collect densitometric data, signal intensity was measured by spot densitometry tool of AlphaEaseFC software (Alpha Innotech Corp, USA). For qRT-PCR and western blotting, the signal intensity (Integrated Density Value, IDV) of KLK8 and MAP2 bands was normalized against signal intensity of glyceraldehyde 3-phosphate dehydrogenase (GAPDH) internal control and plotted as relative density value (RDV). For *in situ* hybridization, signal intensities (IDV/Area) of greyscale images captured by Leica DM 2000 microscope were measured using AlphaEaseFC software and plotted as IDV/area after deduction of negative control and background values

Dendrite growth was analyzed by measuring average length and number of dendrites using Leica LASV4.2 software. Microscopic images from preselected fields (center and upper left corner) were captured, and length and number of dendrites were quantified and expressed as total length and total number of dendrites per treatment group. Average length and number of dendrites was obtained by dividing total length or total dendrites by number of cells within a given field.’

For all above data, initial analysis was by mixed-level generalized linear model to obtain omnibus significance for variables via analysis of deviance (ANODE). For cell culture experiments, technical replication was handled by treating ‘tissue culture plate well’ and ‘experiment’ as random slopes. For mouse experiments, technical replication was handled by treating ‘pool’ as a random slope. We used estimated marginal means (emmeans, Benjamini & Hochberg false discovery rate/FDR adjusted) for pairwise comparisons p values < 0.05 were considered significant. All statistics were done with R statistical environment^100–102^.

Docking analysis was done with the AutoDock Tools (http://autodock.scripps.edu/). The structure description for withanone (https://pubchem.ncbi.nlm.nih.gov/compound/21679027) was obtained from PubChem and for the M1 receptor (http://www.rcsb.org/structure/5CXV) from the RCSB protein data bank.

### Ethics approval

Animal handling and experiments were conducted in accordance with the guidelines of the Institutional Animal Ethical Committee (IAEC), Faculty of Science, Banaras Hindu University Committee for the Purpose of Control And Supervision of Experiments on Animals (CPCSEA), Ministry of Environment, Forest and Climate Change, Govt of India. All experimental protocols involving Swiss albino strain mice were approved by the institutional animal ethical committee of faculty of science of BHU, Varanasi.

## Supplementary information

Supplemental Material-Tables and Figures
