# Supplementary material for: M1 muscarinic receptor is a key target of neuroprotection, neuroregeneration and memory recovery by i-Extract from Withania somnifera
Source: Sci Rep. 2019 Sep 30;9:13990. doi: 10.1038/s41598-019-48238-6 (PMC6769020; doi:10.1038/s41598-019-48238-6)

M1 muscarinic receptor is a key target of neuroprotection, neuroregeneration and memory recovery by i-Extract from *Withania somnifera*.

Arpita Konar, Richa Gupta, Rajendra K. Shukla, Bryan Maloney, Vinay K. Khanna, Renu Wadhwa, Debomoy K. Lahiri, Mahendra K. Thakur

Supplementary Tables 1-6

Crude gels from Fig. 4

Crude gels from Fig. 5

Supplementary Table 1. Percent changes in KLK8 qRT-PCR and WB from saline or scopolamine-treated mice.

Treatment	KLK8 *in situ* RNA				KLK8 qRT-PCR				KLK8 WB				MAP2c WB			
	Change from SA		Change from SC		Change from SA		Change from SC		Change from SA		Change from SC		Change from SA		Change from SC	
	Cortex	Hippoc	Cortex	Hippoc	Cortex	Hippoc	Cortex	Hippoc	Cortex	Hippoc	Cortex	Hippoc	Cortex	Hippoc	Cortex	Hippoc
SA	+0%	+0%	+24%	[§]+285%	+0%	+0%	[§]+38%	[§]+251%	+0%	+0%	[§]+94%	[§]+300%	+0%	+0%	[*]+60%	[*]+188%
SC	−19%	[*]−74%	+0%	+0%	[*]−27%	[*]−71%	+0%	+0%	[*]−48%	[*]−75%	+0%	+0%	[*]−37%	[*]−65%	+0%	+0%
SC →i-E	+5%	+8%	+30%	[§]+317%	[*]+35%	−7%	[§]+86%	[§]+228%	+33%	−32%	[§]+159%	[§]+174%	[*]+21%	[*]−24%	[*]+93%	[*]+118%
i-E	[*]+57%	[*]+132%	[§]+95%	[§]+794%	[*]+120%	[*]+99%	[§]+204%	[§]+597%	[*]+78%	[*]+200%	[§]+245%	[§]+1100%	[*]+65%	[*]+44%	[*]+164%	[*]+314%
i-E → SC	[*]+30%	[*]+40%	[§]+61%	[§]+441%	[*]+42%	[*]+31%	[§]+96%	[§]+360%	+39%	[*]+134%	[§]+170%	[§]+837%	[*]+15%	+11%	[*]+83%	[*]+220%

[*]Difference significant from SA at $p \le 0.05$.

[§]Difference significant from SC at $p \le 0.05$.

Supplementary Table 2 ANODE of mouse brain treatments vs. regions

**Term**	**KLK8 in situ RNA**		**KLK8 qRT-PCR**		**KLK8 WB**		**MAP2c WB**	
	**χ^2 (df)**	**p**	**χ^2 (df)**	**p**	**χ^2 (df)**	**p**	**χ^2 (df)**	**p**
Treatment	1045.2 (4)	<0.001	571.5 (4)	<0.001	651.2 (4)	<0.001	633.5 (4)	<0.001
Region	822.8 (1)	<0.001	0.1 (1)	0.715	299.2 (1)	<0.001	33.4 (1)	<0.001
Treatment × Region	1004.8 (4)	<0.001	162.0 (4)	<0.001	554.6 (4)	<0.001	245.6 (4)	<0.001

Supplementary Table 3. Percent changes in KLK8 qRT-PCR and WB from saline or scopolamine-treated mice vs M1 receptor agonist or antagonist treatment.

Treatment	KLK8 qRT-PCR				KLK8 Western				MAP2c Western			
	Change from SA		Change from SC		Change from SA		Change from SC		Change from SA		Change from SC	
	Cortex	Hippoc	Cortex	Hippoc	Cortex	Hippoc	Cortex	Hippoc	Cortex	Hippoc	Cortex	Hippoc
SA	+0%	+0%	§+172%	§+775%	+0%	+0%	§+315%	§+877%	+0%	+0%	§+221%	§+195%
M1 ant	*−61%	*−74%	+6%	§+127%	*−70%	*−70%	§+26%	§+196%	−67%	*−72%	+5%	−18%
M1 ag	*+157%	*+146%	§+601%	§+2055%	*+152%	*+148%	§+946%	§+2324%	*+160%	*+115%	§+734%	§+532%
SC	*−63%	*−89%	+0%	+0%	*−76%	*−90%	+0%	+0%	−69%	*−66%	+0%	+0%
M1 ag → SC	*+121%	*+92%	§+501%	§+1582%	*+113%	*+94%	§+786%	§+1796%	*+120%	+23%	§+606%	§+263%
i-E → SC	*+87%	*+99%	§+409%	§+1644%	*+89%	*+101%	§+686%	§+1862%	*+86%	*+51%	§+498%	§+344%
M1 ant → i-E → SC	+0%	*−69%	§+173%	§+174%	+7%	*−64%	§+343%	§+247%	+3%	*−73%	§+230%	−20%
i-E	*+223%	*+171%	§+780%	§+2272%	*+220%	*+171%	§+1230%	§+2547%	*+227%	*+163%	§+950%	§+674%
M1 ant → SC	−9%	*`−74%	§+148%	§+124%	−13%	*−73%	§+260%	§+165%	−17%	*−84%	§+166%	−53%

*Difference significant from SA at $p \leq 0.05$.

§Difference significant from SC at $p \leq 0.05$.

Supplementary Table 4 ANODE of mouse brain treatments, including M1 agonist and antagonist, vs. brain regions

**Term**	**KLK8 qRT-PCR**		**KLK8 WB**		**MAP2c WB**	
	**χ^2 (df)**	**p**	**χ^2 (df)**	**p**	**χ^2 (df)**	**p**
Treatment	1627 (8)	<0.001	1845 (8)	<0.001	267 (8)	<0.001
Region	50 (1)	<0.001	55 (1)	<0.001	169 (1)	<0.001
Treatment × Region	410 (8)	<0.001	445 (8)	<0.001	288 (8)	<0.001

Supplementary Table 5. Gavage and Injection Contents for data in Fig. 4

**Treatment**	**Control**	**Scop.**	**Scop. → i-Extract**	**i-Extract**	**i-Extract → Scop.**
**Gavage**	0.5% DMSO	0.5% DMSO	i-Extract in 0.5% DMSO	i-Extract in 0.5% DMSO	i-Extract in 0.5% DMSO
**Injection**	0.9% saline	Scop. In 0.9% Saline	Scop. in 0.9%saline	0.9%saline	Scop. in 0.9%saline
**Order of drugs and separation time**	0.9% saline<br>**1h**↓<br>0.5%DMSO	Scop in 0.9% saline<br>**1h** ↓<br>0.5%DMSO	Scop in 0.9% saline<br>**1h** ↓<br>i-Extract in 0.5% DMSO	0.9% saline<br>**1h**↓<br>i-Extract in 0.5% DMSO	i-Extract in 0.5% DMSO<br>**1h**↓<br>Scop in 0.9% saline

Table lists contents of gavage and injection treatments given to animal groups as portrayed in Fig. 2. Note that multiple substances were not given simultaneously but were separated by hours as specifically described in main text.

Supplementary Table 6. Gavage and Injection Contents for data in Fig. 5

**Treatment**	**Control**	**Antagonist**	**Agonist**	**Scop.**	**Agonist → Scop.**	**i-E → Scop.**	**Antagonist → i-Extract → Scop.**	**i-Extract**	**Antagonist → i-Extract**
**Gavage**	0.5% DMSO	0.5% DMSO	0.5% DMSO	0.5% DMSO	0.5% DMSO	i-Extract in 0.5% DMSO	i-Extract in 0.5% DMSO	i-Extract in 0.5% DMSO	i-Extract in 0.5% DMSO
**Injection**	saline	Antagonist in saline	Agonist in saline	Scop. in Saline	Agonist + Scop. In saline	Scop. in saline	Antagonist + Scop. in saline	Saline	Antagonist in Saline
**Order of drugs and separation time**	0.9% saline **1h**↓ 0.5%DMSO	Antagonist in 0.9% saline **1h**↓ 0.5%DMSO	Agonist in 0.9% saline **1h**↓ 0.5%DMSO	Scop in 0.9% saline **1h** ↓ 0.5%DMSO	Agonist in 0.9% saline **2h**↓ Scop in 0.9% saline **1h** 0.5%DMSO	i-Extract in 0.5% DMSO **1h**↓ Scop in 0.9% saline	Ant in saline **1h**↓ i-Extract in 6% DMSO **1h**↓ Scop. in saline	0.9% saline **1h**↓ i-Extract in 0.5% DMSO	Ant in saline **2h** ↓ i-Extract in 6% DMSO

Table lists contents of gavage and injection treatments given to animal groups as portrayed in Fig. 3. Note that multiple substances were not given simultaneously but were separated by hours as specifically described in main text.

Fig. 4B gels

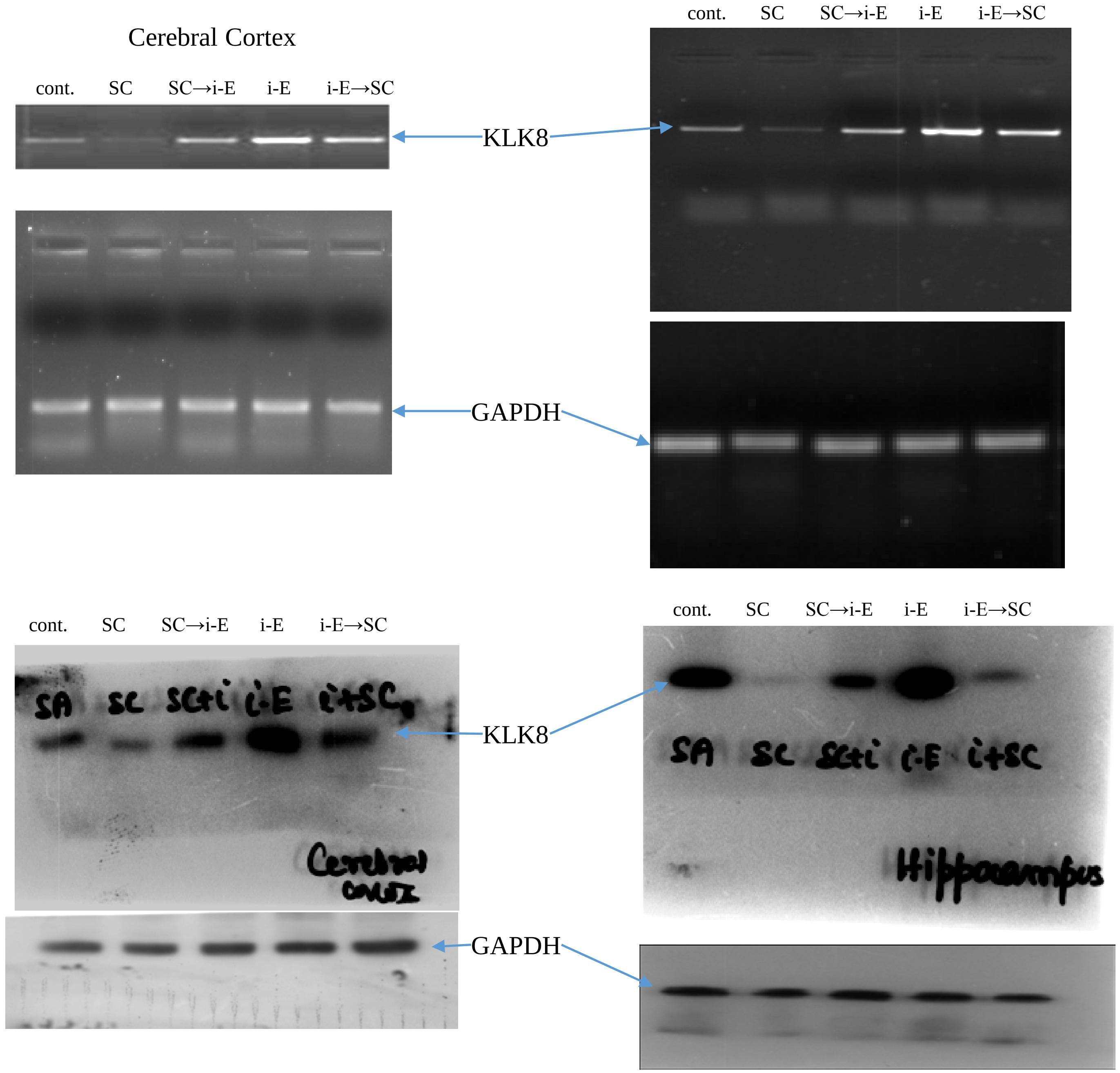

Fig. 4C gels

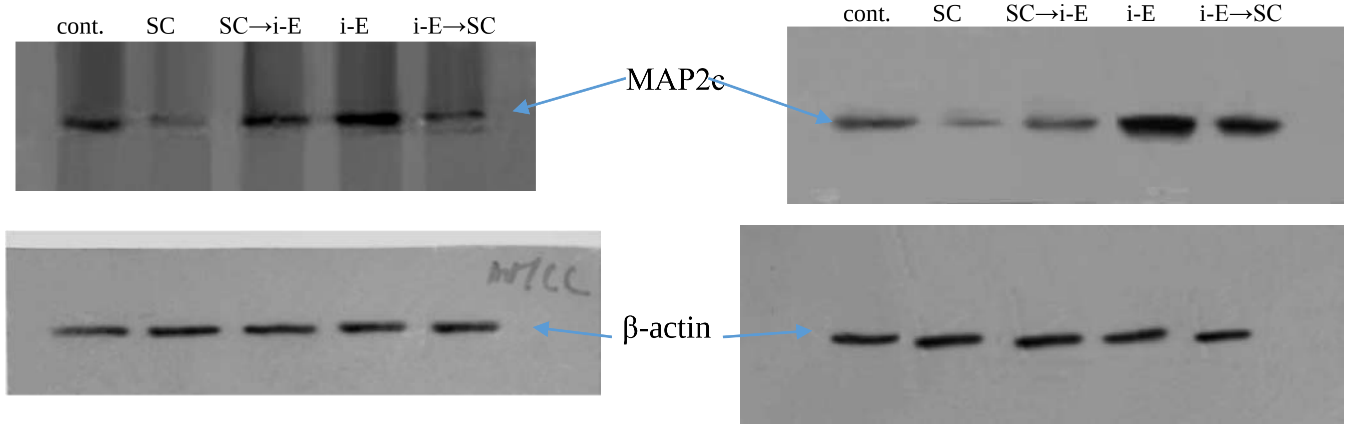

Fig. 5A gels

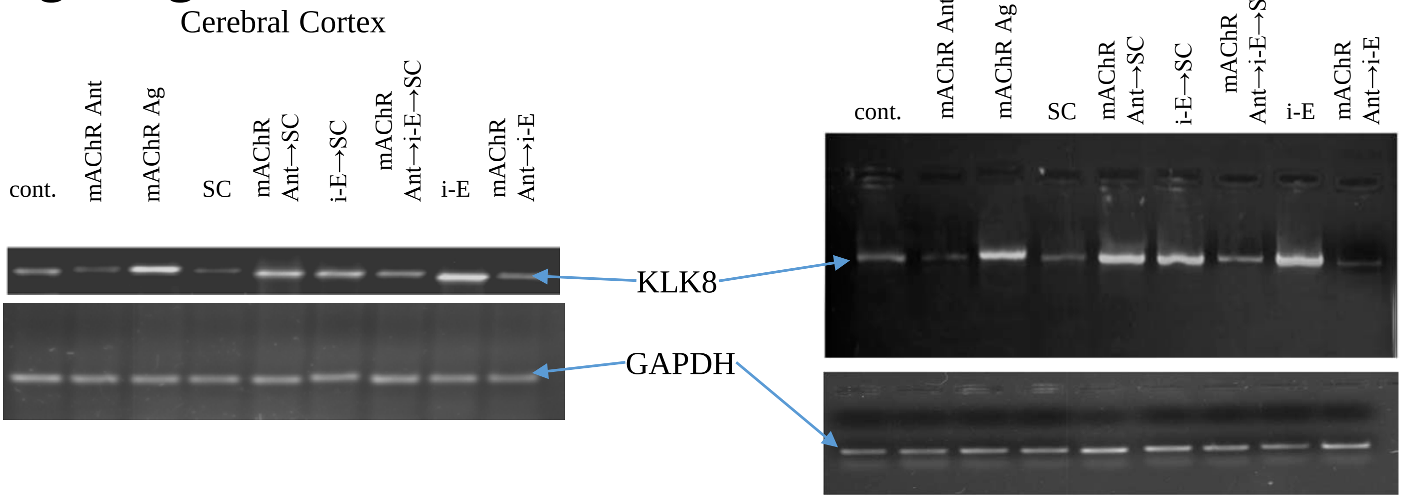

Fig. 5B gels

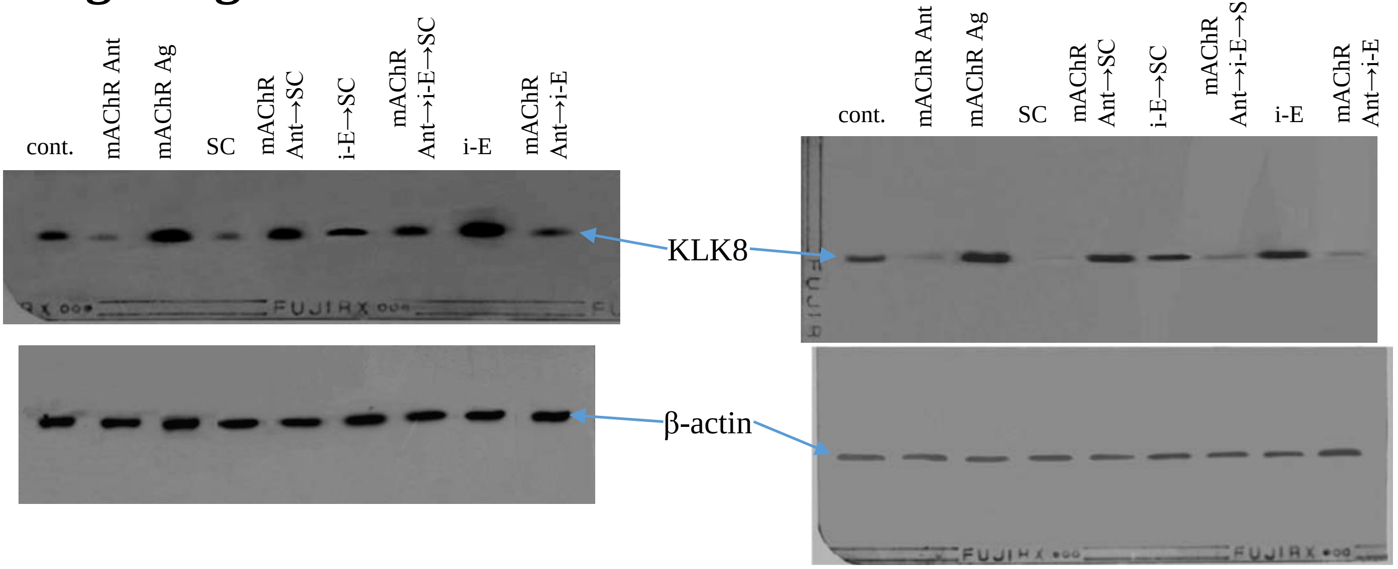

Fig. 5C gels

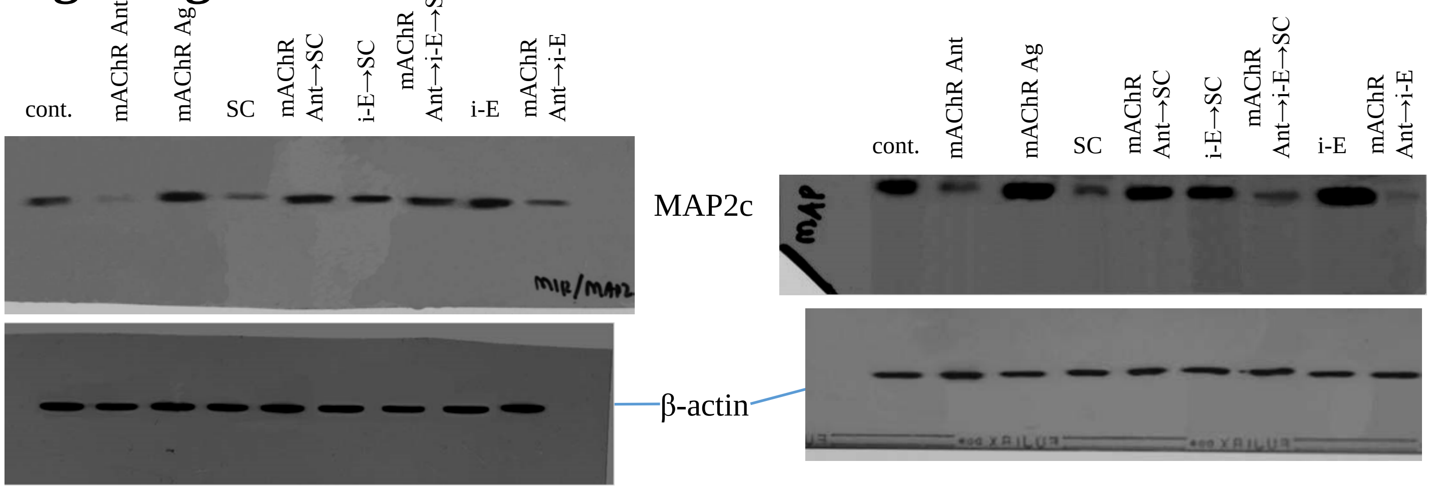

Supplement: Supplementary file 1 — Supplemental Material-Tables and Figures [file 41598_2019_48238_MOESM1_ESM.pdf]
